# Impact of duplicate CT scan rate after implementation of transfer image repository system at a level 1 trauma center

**DOI:** 10.1007/s10140-017-1575-6

**Published:** 2018-01-12

**Authors:** Charles W. Sheppard, Amy L. Groll, Cindy L. Austin, Simon J. Thompson

**Affiliations:** 1 Department of Emergency Medicine, Mercy Hospital Springfield, 1235 E Cherokee St, Springfield, MO 65804 USA; 2 Emergency Department, Mercy Hospital Springfield, 1235 E Cherokee St, Springfield, MO 65804 USA; 3 Trauma and Burn Research, Mercy Hospital Springfield, 1235 E Cherokee St, 7H, Springfield, MO 65804 USA

**Keywords:** CT/MRI, Trauma, Imaging, Critical care transport, Transfer image repository, Duplicate CT, Computed tomography

## Abstract

**Purpose:**

The regionalization of trauma in the USA results in frequent transfers of patients from a primary hospital ED to a higher level trauma facility. While many hospitals have a Picture Archive Communication System (PACS) which captures digital radiological images, these are often not available to the receiving institution resulting in duplicate imaging. The state of Arkansas instituted a trauma image repository (TIR) in July 2013. We examined whether implementation of this repository would impact CT scan duplication in the trauma system.

**Methods:**

This was a retrospective analysis of trauma patients transferred from outlying hospitals in Arkansas and Missouri to a single level 1 trauma hospital in Missouri between July 2012 and June 2015. We compared the duplicate CT rate for patients transferred from Arkansas and Missouri hospitals before and after the repository was implemented for Arkansas.

**Results:**

Prior to implementation (July 2012–June 2013) of Arkansas TIR, duplicate CT rates were similar for patients transferred from Arkansas (11.5% ± 2.8) or Missouri (16.3% ± 7.5). Following implementation (July 2013–June 2014), the duplicate CT rate for patients transferred from Arkansas was significantly lower (Arkansas = 10.1% vs. Missouri 16.2%; CI 95%, *p* = 0.02), and significance continued (Arkansas = 9.0% vs. Missouri = 17.8%; CI 95%, *p* = 0.02) during follow-up (July 2014–June 2015).

**Conclusion:**

Fewer patients received duplicated scans within the Arkansas as compared with the Missouri-based trauma referral systems regardless of Injury Severity Scores (ISS). Our findings suggest that TIR adoption coupled with PACS improved transferability of radiographic studies and could improve patient care while reducing costs in trauma transfers.

## Introduction

The regionalization of trauma in the USA [1], Canada [2], Europe [3], and other parts of the world [4, 5] results in frequent transfers of patients from a primary hospital emergency department to a higher level trauma facility [6]. Currently, most hospitals in the USA have a Picture Archive Communication System (PACS) which captures digital radiological images providing convenient access to images from within one hospital, or a localized group of affiliated hospitals [7]. Initially, the benefit (reviewed by Becker and Arenson 1994 [8]) touted by the introduction of PACS was a reduction in radiation exposure and cost, due to the reduced need to retake the same images [9]. Although the implementation of PACS proved helpful within intra-hospital transfers, problems remained for inter-hospital transfers since images were often not available to the receiving institution, once again resulting in duplicate patient imaging [10].

A review of the literature indicates a vast range of duplicate computed tomography (CT) averages when trauma patients are transferred into a higher level trauma hospital (28–91%; [3, 4, 11–14]). According to Chwals et al. (2008), approximately 50% of trauma patients receive at least one CT at the primary hospital before being transported to the tertiary hospital [14]. Not only does the lack of readily available images impact the timeliness of critical patient care but also the necessary time for the actual CT scanning procedure. Research has shown the average length of time for a CT procedure is approximately 22 min [15]; however, a more recent report found that trauma patients undergoing CT procedures actually had an average increased length of stay (LOS) of 90 min at the primary hospital before transfer [16].

Prompt patient management is a mainstay of trauma care [17]; thus, any unnecessary repeat tests may delay appropriate treatment. Trauma surgeons have long recognized the so-called golden hour, the critical period of time to begin definitive treatment of patients who have suffered serious trauma [18]. The American College of Surgeons Committee on Trauma has recognized that within this golden hour, the aim of resuscitation for these patients is to achieve respiratory and hemodynamic stabilization [17]. It has been shown that treatment delays from prehospital care to in-hospital procedures, such as prolonged radiographic examinations times, can have a deleterious effect on the patient [19].

**Fig. 1:**
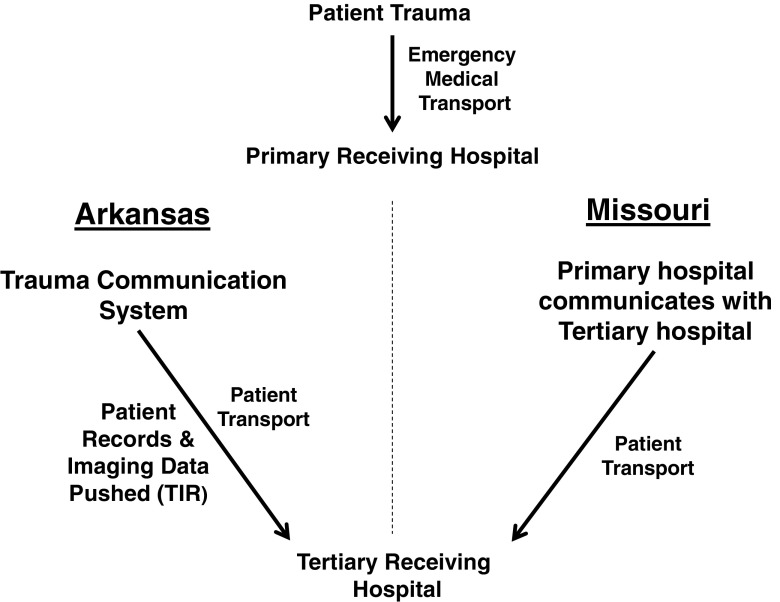
Flow from patient trauma through to the tertiary hospital

Thus, reducing duplicate CTs could minimize patient delay. One way to do this would be to enable tertiary hospitals access to the primary hospitals PACS. Liepert et al., 2014, found a 33% reduction in duplicate CT scans when tertiary hospitals had access to primary receiving hospital CT scans [20]. Implementation of statewide radiological image repositories has been shown to reduce the following: repeat CT scans, significant costs, radiation exposure, and LOS in the ED for patients with less complex injuries [21]. The Arkansas trauma image repository (TIR) is a portal through which CT images can be sent from the primary receiving hospital making the CT information available for upload into the tertiary transferring hospital’s PACS (Fig. 1). The purpose of this study was to compare two different states image handling systems, one with a TIR and one without, for trauma patient transfers to a single hospital utilizing rate of duplicate CT scans as a patient benefit metric.

## Methods

### Setting

The primary hospital is the originating hospital where the patient initially received treatment. The tertiary hospital is the referral hospital where the patient was transported for more specialized care. In this study, the tertiary hospital is a single level 1 trauma center which receives trauma patients via transfer from hospitals in either Arkansas or Missouri (Fig. 1) after initial stabilization. These hospitals utilize a digital PACS to record and view radiological images. Implementation of the Arkansas image repository began July 2013 [22] within the tertiary hospital. In order to evaluate the change in duplication rates over time, data was analyzed a year before and after the image repository implementation.

### Sample

Between July 2012 and June 2015, 2460 patients were transferred from outlying hospitals in Arkansas and Missouri into a single tertiary hospital (for a demographics overview, see Table 1). The total number of trauma transfer patients including a CT = 1476, with the total number of duplicate CT scans, is 194 (13.1%). Patients who did not receive a CT scan from the primary hospital or < 18 years old were excluded from the study.

**Table 1:** Data averaged across the 3 years

		Arkansas	Missouri
Demographics	Patient transfers including at least one CT (*n*)	853	623
	Age (year), Avg. (± SD)	45.3 (± 25.7)	51.7 (± 25.5)
	Female (sex), *n* (%)	288 (33.8%)	236 (37.8%)
	Total primary receiving hospitals (*n*)	28	36
	Most frequent trauma level (1–3) primary hospital, level (overall %)	3 (87.8%)	3 (77.3%)
	Avg. injury severity score (± SD)	10.2 (± 8.6)	10.3 (± 7.0)
Injury etiology	Motor vehicle (including motorcycles), *n* (%)	272 (31.9%)	225 (36.1%)
	Pedestrian, *n* (%)	21 (2.5%)	8 (1.3%)
	Fall, *n* (%)	289 (33.9%)	261(41.9%)
	Gunshot, *n* (%)	27 (3.2%)	13 (2.1%)
	Stabbing, *n* (%)	13 (1.5%)	14 (2.2%)
	Other, *n* (%)	231 (27.1%)	102 (16.4%)
	Total CT scans (*n*)	2360	1207
	Avg. CT scans per person	2.8	1.9
	ICD-9 codes, *n* (%)		
	87.03	678 (28.7%)	381 (31.6%)
	87.41	367 (15.6%)	220 (18.2%)
	88.01	409 (17.3%)	247 (20.5%)
	88.38	906 (38.4%)	359 (29.7%)

### Data analysis

This retrospective analysis examined CT duplication rates using a method previously described by Mohan et al. (2010) [11]; study data was extracted from the tertiary hospital’s trauma registry. The number of patients with a duplicate CT within 24 h of arrival was identified by matching exact exam types utilizing ICD-9 codes (CT head, 87.03; CT thorax, 87.41; CT abdomen, 88.01; CT other, skeletal 88.38). Duplication of a CT was defined as a patient who had at least one CT scan at an outlying primary hospital and then transferred to the tertiary hospital receiving the exact same CT scan while in the emergency department or within 24 h of arrival.

Duplication rates were calculated for patients meeting the duplicate CT definition and inclusion criteria. These rates were calculated as one per patient not per CT, despite the number of duplicate CTs a multiple injury patient may have received. For example, a trauma patient with three duplicate CTs (head, thorax, and abdomen) was only counted as one duplicate CT.

The data was divided into three phases to demonstrate impact of TIR implementation and measure its sustainability over a 2-year period: pre-TIR implementation year (July 2012–June 2013), implementation year (July 2013–June 2014), and follow-up year (July 2014–June 2015) to show any continued maintainable effect.

### Statistics

Due to the seasonal differences in trauma patient transfer volume, duplicate CT percentage was calculated [11] per quarter, expressed as average percentile across 12 months ± standard deviation. A two-tailed Student’s *t* test was used to compare CT duplication rates for each study year for trauma patients transferred from Arkansas and Missouri, respectively, in a year; results were accepted as significant when the CI 95%, *p* ≤ 0.05.

The study received approval from the Mercy Institutional Ethics Review Board. For this type of study, formal consent is not required.

## Results

Averaged across the whole timeframe of this study (shown in Table 1), Arkansas and Missouri patients have similar demographics; however, trauma patients transferred from Arkansas received an average of 2.8 CT scans per person, as compared to those transferred from Missouri who received an average of 1.9 CT scans per person.

Exploring the data within the yearly phases of this study, the CT duplication rate decreased each year after TIR introduction for patients transferred from Arkansas; in year 1, the duplication rate was 11.5% ± 2.8, and the two subsequent years showed reductions, 10.1% ± 3.4 (year 2) and 9.0% ± 2.2 (year 3). For those patients transferred from Missouri, there was little change from year 1 (16.3% ± 7.5) to year 2 (16.2% ± 2.4), followed in year 3 by a slight increase to 17.8% ± 3.4.

**Fig. 2:**
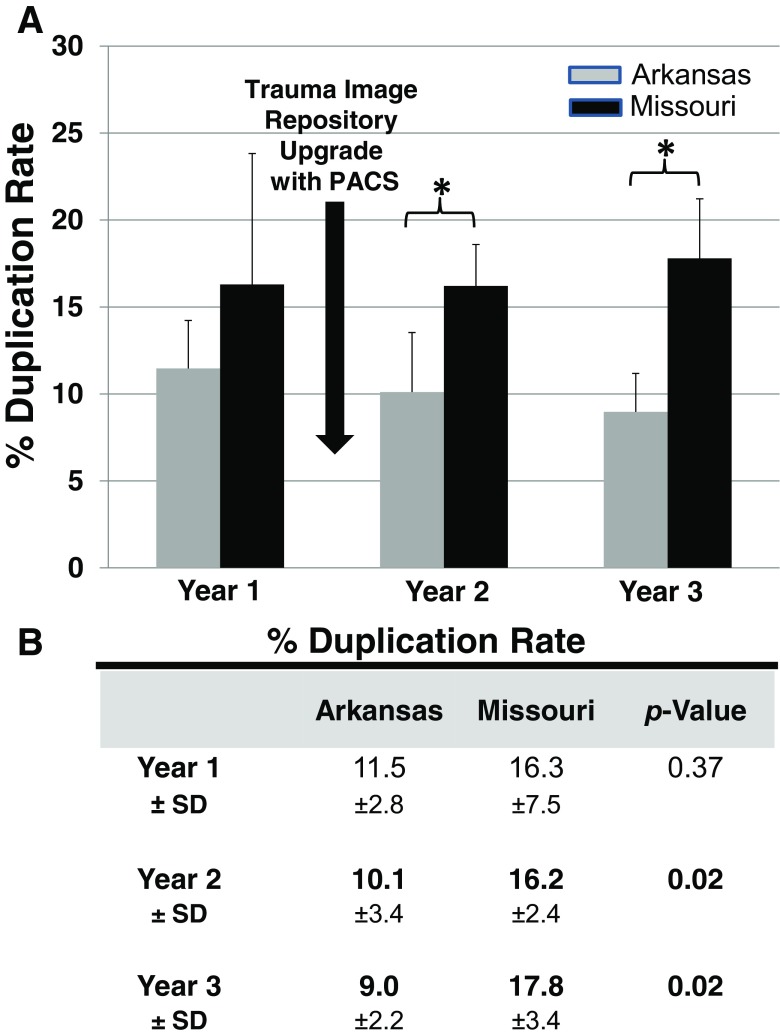
Comparing the originating state transfers in relation to duplicate CT scans into a level 1 trauma facility. Data was separated according to the originating hospital state (Arkansas; Missouri) then quarterly data averaged across a year (years 1, 2, and 3). Duplicate CT percentage was calculated by taking the number of patients with duplicate CTs in a quarter and dividing by the total number of trauma patient transfers that include a CT scan in that quarter. **a** Error bars are standard deviation; the asterisk symbol indicates a two-tailed *p* value ≤ 0.02. Black arrow denotes date Arkansas trauma repository upgrade was integrated into the SW Missouri trauma level I center PACS. **b** *p* values ≤ 0.05 were considered significant

Prior to implementation of the Arkansas TIR, there was no significant difference in duplication rate between Missouri and Arkansas transfer patients. From year 2, comparing the duplicate CT rate between the states, Arkansas transfers were significantly lower than those in Missouri for each of the subsequent years after the TIR was implemented (*p* ≤ 0.02; Fig. 2).

## Discussion

From a purely hypothetical perspective, the implementation of a TIR and trauma communication system (see Fig. 1), by reducing unnecessary repeat CTs should have a positive effect on transfer time, patient care costs, and radiation exposure. This is corroborated in the literature, where PACS implementation between hospitals shows reduction in the duplicate CT rates in trauma transfers [13, 23]. Furthermore, the literature shows reducing duplicate CT rates that reduces patient exposure and care costs [3]. Thus, the goal for this study was to determine whether implementation of a state-level PACS-like system within a trauma paradigm would impact duplicate CT scan rates.

First, when compared between the two states, Arkansas patients transferring from primary hospitals received more CT scans per person than those transferring from Missouri primary hospitals. However, Arkansas patients received fewer CT duplications than those patients from Missouri. Secondly, when examined by year, fewer Arkansas patients received duplicate CT scans after the integration into our hospital PACS (June 2013). Furthermore, comparing the Arkansas and Missouri transfer patients, we show the implementation of the TIR, again, introduces a point that separates the patient CT duplication data (Fig. 2) that is further solidified during year 3.

Although not examined, one plausible factor between the various hospital systems each has to follow is the state regulations for trauma. The Missouri state trauma system inception was in the 1990s [24], but the Arkansas state trauma system began in 2009 [25]. The advantage for the later Arkansas inception date allowed inclusion of web sharing-based applications (TIR) into the state regulations, thus requiring greater interoperability between the various hospitals chosen imaging file types for compatibility with the TIR.

## Conclusion

In conclusion, these findings suggest that the adoption by the tertiary hospital of a state-level TIR eventually led to a 22% reduction in CT duplication when comparing Arkansas transfer patients. When comparing between the Arkansas and Missouri transfer patients, there is a 50% reduction in CT duplication.

## Limitations

This study includes data compiled from only a single center, lacking comparison to other hospitals and inability to warrant whether repeat CT was performed for clinically validated reasons.

There are several issues that may impede data transfers between primary and receiving hospitals: radiologist delay in reading CT scan and uploading report and technical problems due to incompatibility, damaged, lost, or unreadable CDs [26].
